# Building Facade Reconstruction by Fusing Terrestrial Laser Points and Images

**DOI:** 10.3390/s90604525

**Published:** 2009-06-09

**Authors:** Shi Pu, George Vosselman

**Affiliations:** International Institute for Geo-Information Science and Earth Observation (ITC), Hengelosestraat 99, 7500 AA Enschede, The Netherlands; E-Mail: vosselman@itc.nl

**Keywords:** building reconstruction, terrestrial laser scanning, data fusion, texturing

## Abstract

Laser data and optical data have a complementary nature for three dimensional feature extraction. Efficient integration of the two data sources will lead to a more reliable and automated extraction of three dimensional features. This paper presents a semiautomatic building facade reconstruction approach, which efficiently combines information from terrestrial laser point clouds and close range images. A building facade's general structure is discovered and established using the planar features from laser data. Then strong lines in images are extracted using Canny extractor and Hough transformation, and compared with current model edges for necessary improvement. Finally, textures with optimal visibility are selected and applied according to accurate image orientations. Solutions to several challenge problems throughout the collaborated reconstruction, such as referencing between laser points and multiple images and automated texturing, are described. The limitations and remaining works of this approach are also discussed.

## 1. Introduction

Realistic 3D building facade models are beneficial to various fields such as urban planning, heritage documentation and computer games. A manual reconstruction process can be rather time-consuming and inaccurate. The operators need to interpret the reference data, draw boundary represented models using some 3D modeling software (3DS Max for example), select and undistort all the texture parts, and finally apply corresponding textures to each face of the model. Creating building facade models for a whole city requires considerable work, therefore for decades much research has been dedicated to the automation of this reconstruction process.

Nowadays a number of facade reconstruction approaches are available, which are based either on close range images [1, 2] or terrestrial laser data [3, 4, 5]. Close range images have been commonly used for building facade reconstruction for decades because they contain plentiful optical information which can be easily acquired. However, there are still few automated approaches that are able to extract 3D building structures from 2D images. The lack of automation in image based approaches can be explained by the difficulties in image interpretation and image-model space transformation. Specifically, factors like illumination and occlusion can cause considerable confusion for machine understanding and a number of conditions (relative orientation, feature matching, etc.) need to be accurately determined to transfer image pixels to 3D coordinates. In recent years, terrestrial laser scanning data has been proven as a valuable source for building facade reconstruction. The point density of stationary laser scanning in urban areas can be up to hundreds or thousands of points per square meter, which is definitely high enough for documenting most details on building facades. The latest mobile laser scanning platforms like Lynx and Streetmapper can also provide quite dense point clouds during high speed driving. Laser data based reconstruction approaches face the challenging task of extracting meaningful structures from huge amount of data. Besides, the laser beam doesn't contain any color information, so combination with optical data is inevitable if texturing is required.

Much research [6, 7] suggests that laser data and optical data have a complementary nature to 3D feature extraction, and efficient integration of the two data sources will lead to a more reliable and automated extraction of 3D features. In [8], the normalized difference vegetation indices (NDVI) from multi-spectral images and the first and last pulses from airborne laser data, are fused for classifying vegetation, terrain and buildings. [9] integrates airborne laser data and IKONOS images for building footprints extraction. Like in [8], fusion of the two data types benefits the classification of building regions. In addition, the two data types also collaborate in 1) the feature extraction stage, where the building boundaries are designated in the image according to the locations of classified building laser points; and 2) the modeling stage, where the linear features around building boundary from the images and model-fitted lines from laser points are combined together to form a initial building footprint. In the building facade reconstruction process presented in [3], close-range images are used for texturing the building facade models generated from terrestrial laser point clouds. After distinguishing foreground (occlusions) laser points from background (walls) laser points by histogram analysis, the foreground laser points are projected onto close-range images so that the projected image regions will not be mapped to background mesh for texturing.

This paper presents a reconstruction method which aims at generating photorealistic building facade models by efficient fusion of terrestrial laser data and close range images. Section 2 first gives an overview of the presented method. Then different stages of the reconstruction process are elaborated from Section 3 to 5. Section 3 explains the registration algorithms for referencing the laser data space and the image space. Section 4 explains how images can be used to refine the building models generated from laser points. The optimal texturing strategies are given in Section 5. Section 6 examines the applicability of the method with two test cases. Some conclusions are drawn in the final section.

## 2. The Reconstruction Approach

### 2.1. Overview

**Figure 1.**
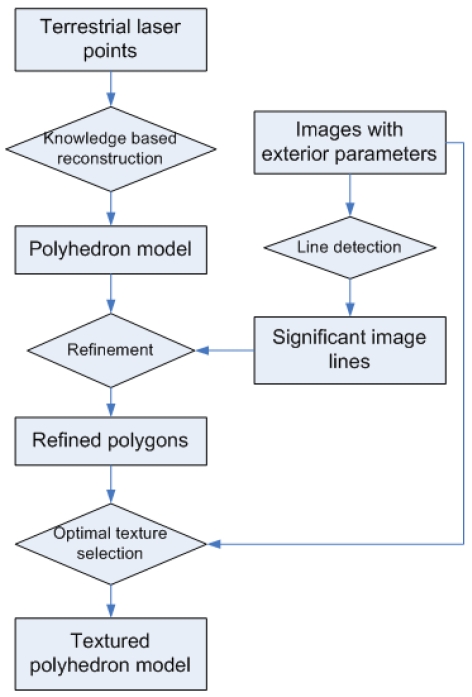
Building reconstruction process.

Line extraction from images is very accurate, while laser points are more suitable for extracting planar features. The mutual independent advantages motivate this combined reconstruction method. The overall process of the presented method is illustrated in Figure 1. In general, a building facade's general structure is established with the planar features extracted from laser data, then image features are introduced to refine the model details. In the preprocessing stage, the exterior orientations of the images are calculated using a series of semi-automated operations. Then the planar features are extracted from terrestrial laser points and modeled as an initial polyhedron model using a previous method presented in [5]. Because of the limitations of modeling algorithm, the initial model is still not accurate for texturing purpose, therefore significant line features extracted from images are compared with the initial model's edges, and necessary refinements are made according to the image lines. Finally in the texturing stage, textures of different model faces are selected automatically from multiple images to ensure the optimal visibility. Texture errors caused by occlusions in front of a wall are also removed by analyzing the locations of the wall, the occlusions and the camera position.

### 2.2. Previous Work

Our previous work of building facade reconstruction from terrestrial laser data is briefly introduced here because of its strong relevance for this paper. The approach first defines several important building features (wall, window, door, roof, and protrusion) based on knowledge about building facades [5]. Then the laser point cloud is segmented into planar segments using the region growing algorithm [10], and each segment is compared with building feature constraints to determine which feature this segment represents. The feature extraction method works fine for all facade features except for windows, because there are usually insufficient laser points reflected from window glass. Instead, windows are located from the holes on the wall features. Finally, outline polygons are fitted from feature segments, and combined to a complete polyhedron model. A significant advantage of this approach is that semantic feature types are extracted and linked to the resulting models, so that i) it is possible to get faster visualization by sharing the same texture for same feature type; ii) polygons can be associated with various attributes according to its feature type.

**Figure 2.**
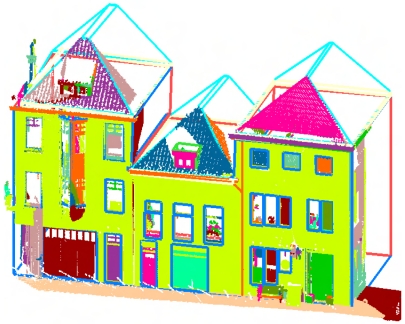
A reconstructed building facade model, shown together with segmented laser points (color of point indicates segment; color of line indicates feature type).

Figure 2 shows a building facade model which is reconstructed with the above approach. Most facade features are successfully extracted and modeled. However, if take a close look, it is easy to identify several mistakes from the model. By analyzing more models, two main reasons for the modeling errors are deduced. They are:

- Limitations of outline generation method. For example, side wall's eave can ”attract” the side boundary edges of the facade, and result in a slight wider polygon in horizontal direction. The almost vertical or horizontal edges are forced to be vertical or horizontal; however, this is not always beneficial.
- Poor scanning quality. Due to the scanning strategy of stationary laser scanner, complete scanning of a scene seems impossible. There are always some parts which contain very sparse laser points, because of the visibility is poor to all scan stations. Occluded zones without any laser points are also usual in laser point clouds. The lack of reference laser information leads to gaps in the final model. Sometimes these gaps are removed using knowledge, but this is not as accurate as data driven modeling.

## 3. Registration

Registration between different laser scans is required after stationary laser scanning to obtain a laser point cloud of the complete scene. There are a number of Iterative Closest Points (ICP) based algorithms available to fulfill this task [11, 12, 13], and this is not discussed here.

**Figure 3.**
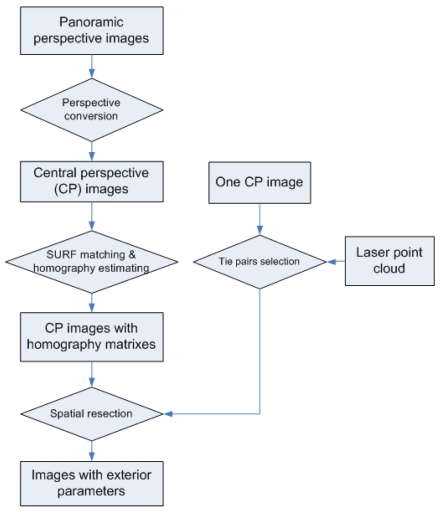
Registration between laser points and images.

In this section, a “laser points to image” registration process is presented (see Figure 3) which aims at calculating accurate exterior orientations of images. This process, also referred to as spatial resection, is necessary before information from image and laser points can be merged for building facade reconstruction. The key to the spatial resection problem is to choose enough tie pairs between laser points and image pixels for collinearity equations. Although automated corresponding selection via Scale-invariant Feature Transform (SIFT, [14]) matching between laser range images and optical images are reported, we find that satisfactory results are difficult to achieve in practice. The laser range images are colored by the reflectance strength of the laser beams, while the colors of optical images are determined by the strength of visible lights, shadows, and the own color of objects. The two kinds of light have rather different natures (compositions, wavelength, etc.), and their exposure directions are also different. These differences make it difficult to achieve automated and reliable feature matching between range images and optical images. Instead, manual selection of tie pairs is adopted to provide inputs for spatial resection of a single image. When there are multiple images available for the same facade, the homographically (also referred as projective transformation) matrixes between images are calculated by image matching. Once the manually selected image pixels in one image are homographic transformed to other images, the spatial resection can be automatically done for all images.

### 3.1. Perspective Conversion

A kind of panoramic images called Cyclorama [15] is used in this research. The Cyclorama images are created from two fisheye images with a field of view of 185 degrees each. The camera is turned 180 degrees between the two shots. Full sphere of image data is stored in a Cyclorama image with 4,800 by 2,400 pixels, corresponding to 360 degree in horizontal direction and 180 degrees in vertical direction. Thus, on both directions the angular resolution is 0.075 degree per pixel. With the integrated GPS and IMU devices, all Cyclorama images are provided with north direction aligned at x = 2,400 and horizontal plane aligned at y = 1,200. The acquisition positions of Cycloramas are also provided from GPS, but they are not very reliable.

In order to be used for facade modeling and texturing, the Cyclorama images need to be converted to central perspective first. The equiangular projection of the fisheye camera model is described in [16]. The projection from panoramic perspective to central projective can be understood as projecting a panoramic sphere part to an assumed plane. First, two lines are created by connecting the image acquisition point (perspective center) with the most left and most right vertices of the initial polyhedron model. The angle of the two lines with north direction derive the longitude boundaries of the region of interest (ROI). In practice it is necessary to widen the ROI to both left and right by a few pixels, because of the errors of the perspective center may cause miss selection of some desired region. Suppose the GPS error is *e* meters, the distance between the acquisition position and a building facade is *d* meters, and the angular resolution is *r* degree/pixel. The offset *o* of a vertical building edge is between *tan*(*e*/*d*)/*r* and −*tan*(*e*/*d*)/*r* degrees. The ROI need to be widened by *o* pixels to both left and right. The principal point is set on the sphere equator, with middle longitude of the two boundaries. Assuming the perspective centers coincide in both perspectives, the pixels inside the ROI are converted from panoramic perspective to central perspective according to the following equations:

(1) $$\alpha =\frac{x_{p}-x_{0}}{r}$$

(2) $$\beta =\frac{y_{p}-y_{0}}{r}$$

(3) $$\tan\alpha =\frac{x_{c}-x_{0}}{f}$$

(4) $$\tan\beta =\frac{(y_{c}-y_{0})\times \cos\alpha }{f}$$

where (*x_p_*, *y_p_*) is the pixel coordinate in panoramic perspective; (*x_c_*, *y_c_*) is the pixel coordinate in central perspective; (*x*_0_, *y*_0_) is the principle point; *r* is the angular resolution; *α* and *β* represent the longitude and latitude of the pixel on the panoramic sphere; *f* is the distance of the panoramic sphere center to the assumed plane, can also be seen as the focal length of the converted central perspective image. With Equations 1 to 4 the unique relation between (*x_p_*, *y_p_*) and (*x_c_*, *y_c_*) can be determined.

### 3.2. Spatial Resection

**Figure 4.**
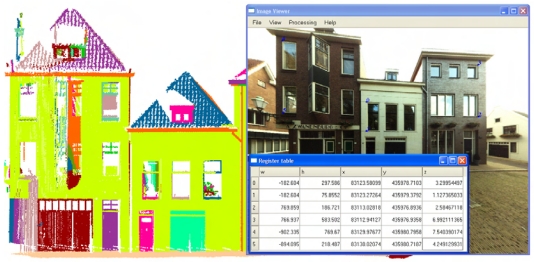
Selecting tie points for spatial resection.

In order to get an unique solution for the six unknown exterior orientation parameters, at least observations of three image control points should be available to form six collinearity equations. Figure 4 illustrates the interface for selecting tie points from a laser point cloud and an image. In the implementation it is required to select at least four tie pairs, with one pair for error checking. If more than four pairs are selected, a least square adjustment is performed to obtain better results.

### 3.3. Relative Orientation

The Cyclorama images are systematically acquired with an interval of a few meters, so a building facade is usually visible from multiple Cyclorama images, and it is possible to choose images with the best visibility for different building parts (see Section 5 for the motivation). It is time-consuming and unnecessary to manually select tie points from all images for spatial resection. There are several feature detection algorithms in image processing field. Sufficient image features from two images can be matched to estimate the homography model between them. In the spatial resection explained in 3.2, the manually selected laser points are tied with image pixels in only one image. However, occurrences of the pixels in other images can be automatically located if the homography models are known. The required tie pairs are therefore generated for each image for spatial resections.

**Figure 5.**
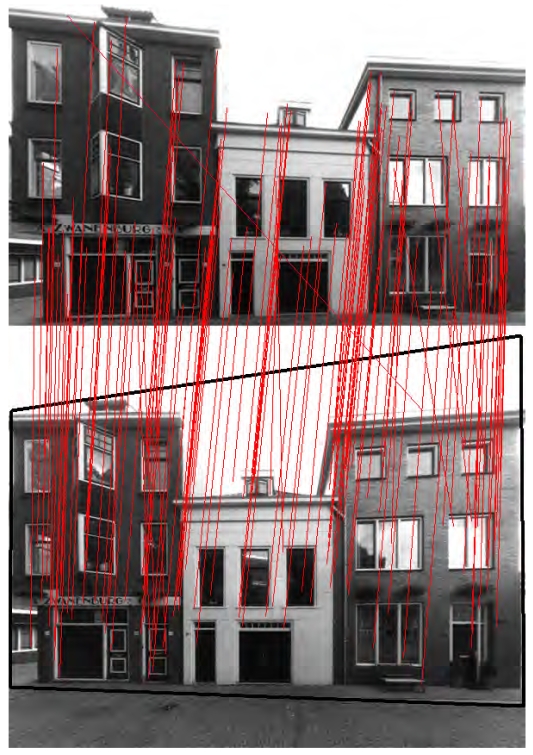
Homography estimation using extracted SURF in two images (red lines: linked SURF pairs; black quadrangle: homographic transformed viewing plane of the upper image).

**Figure 6.**
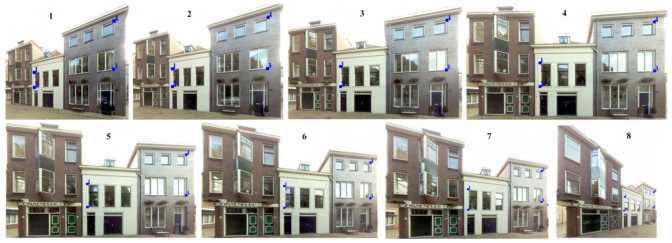
Locating occurrences of the same laser points in different image perspectives.

A scale- and rotation-invariant detector and descriptor called Speed-Up Robust Features (SURF) is used because it can extract satisfactory correspondences between different perspectives, and the processing speed is several times faster than SIFT. A detailed explanation of SURF can be found in [17]. The extracted SURF from two images are compared according to same Laplacian signs and minimal sum of Haar wavelet responses. Then homography matrix between the two images is estimated by applying RANSAC (RANdom SAmple Consensus, [18]) to the SURF pairs. Figure 5 shows the linked SURF pairs (red lines) between two perspectives of a building facade and the homographic transformed viewing plane (black quadrangle). Each image pair with neighboring acquisition positions is processed with the above procedure to calculate their homography matrixes. Figure 6 shows the projections of four laser points in different image perspectives, and the numbers indicate the images' acquisition positions from right to left. The four tie points are manually selected from the image No.5 in Figure 6, and the marks in other images are automatically plotted by multiplying homography matrixes to the tie points' image coordinates in image No.5. Sufficient collinearity equations can be formed for each image using the same 3D object coordinates and different image coordinates, and each exterior orientation is therefore calculated.

## 4. Geometry Refinement

A building model reconstructed from laser points usually contains errors, due to under-sampling of laser scanning and limitations of modeling algorithm. Line extraction from images is very accurate, which can be integrated in the modeling stage to refine the geometry extracted from only laser points. This collaboration is also necessary to achieve better texturing results. The errors in spatial resection result in inaccurate exterior orientations. Even very small inaccuracy can lead to a few pixels' offset at textures' boundaries. “Tuning” between images and model edges before the texturing stage can avoid these inconsistent texturing effects.

### 4.1. Extraction of Significant Lines from Images

**Figure 7.**
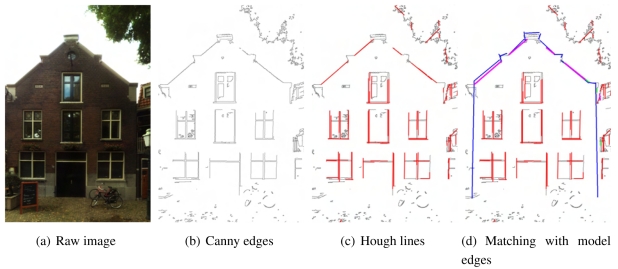
Extracting significant lines from an image (black: Canny edges; red: Hough lines; blue: projections of model edges; green: candidate matches; purple: best matches).

The Canny edge detector algorithm [19] is used for extracting initial line features from images (see Figure 7(a) and Figure 7(b)). Here two threshold parameters should be specified for edge linking and finding initial segments of strong edges. Thresholds set too high can miss important information. On the other hand, thresholds set too low will falsely identify irrelevant information as important. It is difficult to give a generic threshold that works well on all images. In addition to the conventional Canny algorithm, a histogram analysis is made on the image gradients in order to adaptively specify the threshold values. However, factors such as illumination, material, and occlusions still result in many irrelevant edges. In the other hand, some desired edges may not be extracted due to the nature of images. For example, outlines of a wall with very similar color as the surrounding environment will not be detected. Outlines inside shadow areas can hardly be extracted either.

Strong line features are further extracted from Canny edges by Hough transformation (see Figure 7(c)). Because of the unpredicted number of edges resulted from the previous step, a lot of irrelevant Hough line segments may also be generated. To minimize the number of these noise lines, instead of adjusting the thresholds of Hough transformation, all Hough line segments are sorted according to their length, and only a certain number of long ones are kept. This is based on the assumption that building outlines are more the less the most significant edges in an image. The limitations of this assumption are already anticipated before applying to practice. For example, large and vivid patterns on a wall's surface can result in more significant line features than the wall edges.

### 4.2. Matching Model Edges with Image Lines

To match model edges and the image lines for refinement, both should be located either in the 3D model space or 2D image space. Matching in image space is chosen because projecting geometry from 3D to 2D is much easier than determining the 3D coordinates of an image pixel. With the calculated exterior orientation parameters from spatial resection and the focal length, model edges can be projected to the image space according to the collinearity equations (see the blue lines in Figure 7(d)).

Assuming a relatively accurate exterior orientation and the focal length are available, the best matched image Hough line for a model edge is determined in two stages:

1. Candidates of best matching image lines are filtered by their parallelism and distance with the model edge (see the green lines in Figure 7(d)). In other words, the angle between a candidate with the model edges should be smaller than a threshold (5 degree for example), and their distance should also be smaller than a threshold (half a meter for example). Note the actual distance threshold is in pixel, which are also ”projected” from a 3D distance on the wall plane. If the exterior orientation and focal length are perfect, most model edges should coincide very well with a strong image line. However, in practice there may be a small offset and angle between a model edge and its corresponding image line. The optimal angle and distance threshold value are dependent on the quality of the exterior and interior orientations.
2. A best match is chosen from all candidates according to either the collinearity of the candidates or the candidate's length (see the purple lines in Figure 7(d)). It is a common case that a strong line is split to multiple parts by occlusions or shadows. If a number of Hough line segments belong to a same line, this line is set as the best match. If not, the longest candidate is just chosen as the best match.

No spatial index is established in the image space to improve the comparison efficiency, because the search space is already localized to a single building facade, which includes only dozens of edges and Hough lines.

A limitation of this matching method is that it can hardly determine the correct corresponding edge if too many similar line features are within searching range. Simply comparing the geometry properties of position, direction and length are not sufficient in this case. For example, eaves often result in many significant lines and they are all parallel and close to the wall's upper boundary edges. These eave lines can be distinguished if the eave is also reconstructed and included in the facade model, but ambiguity caused by pure color pattern is still difficult to solve.

### 4.3. The Refinement

After matching, most model edges should be associated with a best matched image line. These model edges are updated by projecting to their best matched image line. There are some model edges which don't match any image lines. If no change is made to an edge with its previous or next edge changed, strange shapes like sharp corners and self-intersections may be generated. Therefore interpolations of the angle and distance change from the previous and next edges, are applied to the edges without matched image lines. With these refinement strategies, an original model is updated to be consistent with the geometry extracted from images, and the model's geometry validity and general shape are also maintained.

Finally, the refined model edges in image space need to be transferred back to the model space. We assume the model edges are only moved on their original 3D planes. The collinearity equations are used together with the mathematical equation of the 3D plane to calculate the new 3D positions of all the modified model vertices.

**Figure 8.**
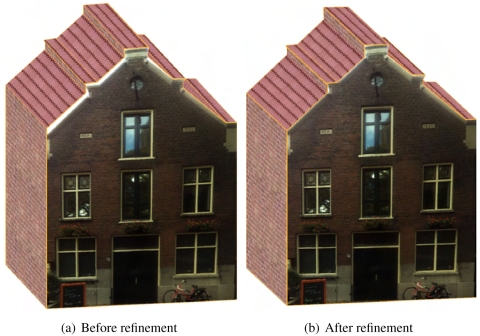
Refining the wall outlines of a building facade.

Figure 8 shows a building facade before and after its wall outlines are refined. It is clearly shown that although the offset between the initial outlines and the image lines are only a few pixels, the texturing effect is very poor due to the contrast between the background's color and the wall's color. With the refinement operation, visualization is significantly improved.

**Figure 9.**
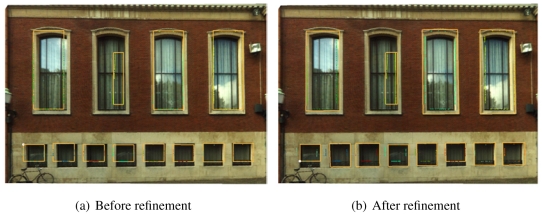
Refining the window outlines of a building facade.

Figure 9 shows another building facade before and after its window outlines are refined. As mentioned earlier, the window rectangles are modeled from the holes on the laser points of the wall. The shapes of holes can be influenced by curtains, window frames and decorations. After refinement with image lines, most windows' boundaries are well corrected according to the image lines. The second left window in the upper row is not improved, because the difference between the initial shape and the actual shape is too large to correlate. There are some remaining errors, such as the first, third and sixth window (from left to right) in the lower row. This is because the parameters of Hough transform are too strict to generate any Hough line for matching.

## 5. Texturing

Texturing makes significant improvement to the visualization of a virtual scene. In addition to the geometric information, correctly textured models will provide extra information in color which are easily understandable. This section concentrates on the texturing strategies for obtaining photorealistic building facade models.

When there is no optical data available for the complete or part of modeling scene, the reconstructed models can be textured by predefined images or just colors. In this research, different parts of the polyhedron model are associated with semantic types. All polygons of wall feature can be textured by a predefined wall picture; roof feature textured by a predefined roof picture; and so on.

When images or videos are available, and the exterior orientation of each image or video frame is known, color information of any facade region in the laser points' space can be extracted from a corresponded image region. When multiple images are available for a building facade, there will also be multiple corresponded image regions for texturing. If textures are only selected from one image, factors like resolution, illumination and occlusion can easily lead to poor texturing. For example, the quality of image areas which are far away from the perspective center can be much worse than the central area. Occluded textures are often selected if the angle between the viewing direction and principle direction is large. Selecting textures with the best visibility from multiple candidate images will make the final model much more realistic.

### 5.1. Optimal Texture Selection

The general assumption for optimal texture selection is that the best texture image *T* for a polygon should be taken directly in front of this polygon. Specifically, there are two considerations for the picking an appropriate texture image:

- The acquisition position of *T* should not be too far away from the polygon, so that the resolution is enough for clear texturing.
- If *L* represents a 3D line decided by the centroid and the normal direction of the polygon, the principle direction of *T* should more or less coincide with *L*.

Taking the two considerations into account, at first all available images are filtered by their distances to the target building facade. Only images taken within a certain distance are kept. Then distances *d* between the image acquisition positions and *L* of each model polygon are computed. The image with minimum *d* to a polygon is assigned to texture this polygon.

**Figure 10.**
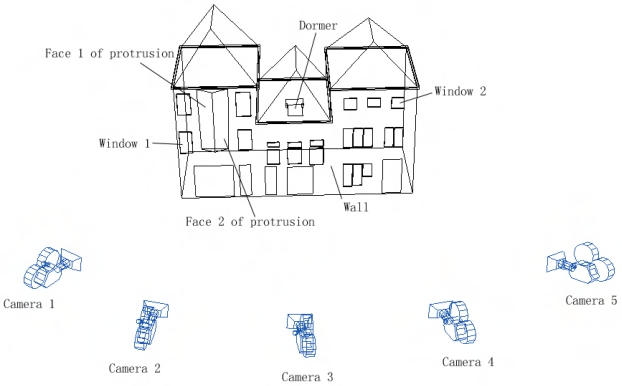
Choosing the optimal texture images for different model parts.

The optimal texture selection strategy as explained above is illustrated in Figure 10. Only the camera positions which are within 15 meters from the building facade are kept as texture candidates. After the operations mentioned above, it is decided that the wall's texture should be selected from the image of camera position 3. It is best to select texture from camera 2 for window 1, and camera 4 for window 2. Face 1 of the protrusion can be textured by the image of either camera 1 or camera 2, while the other face should be textured by images of either camera 3 or camera 4. The dormer should be textured by the image of camera 3.

### 5.2. Occlusion Removal

**Figure 11.**
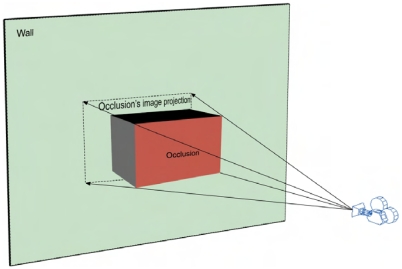
An occlusion and its central perspective projection on a wall.

Images of building facades often include occlusions such as trees, wall protrusions and people walking by (see Figure 11). If the occlusions remain on the background textures, the final visualization effect can be very poor, especially when viewing from a different direction from the image capturing direction.

If the positions of the occlusions are unknown, a voting process can be adopted based on the assumption that occurrence of the foreground color should be less than the background color. For each pixel in the background region, colors of the corresponding pixels in all other images are extracted to form a color histogram, and the most occurrence is believed to be the background color. This method has two main drawbacks: first, the assumption is not necessarily true; second, the computational demand is quite heavy.

In the context of this research, positions of perspective centers, foreground and background are all known, so that the occluded texture regions can be located. Suppose region G is the geometry projection of the occlusion onto the wall (see Figure 11), and region T is the image projection under central perspective. The region of T minus G is textured alternatively by pixels from other perspectives.

## 6. Test Cases

In this section, two test cases are provided to demonstrate the applicability and remaining problems of the presented method. The densities of both laser point clouds are approximately 800 points per square meter. The Cyclorama images are captured with intervals of one meter, so usually any building is visible from more than 10 images. However, the actually used Cyclorama images are resampled to intervals of 3 meter to reduce the processing time.

### 6.1. The Three Joined Houses

**Figure 12.**
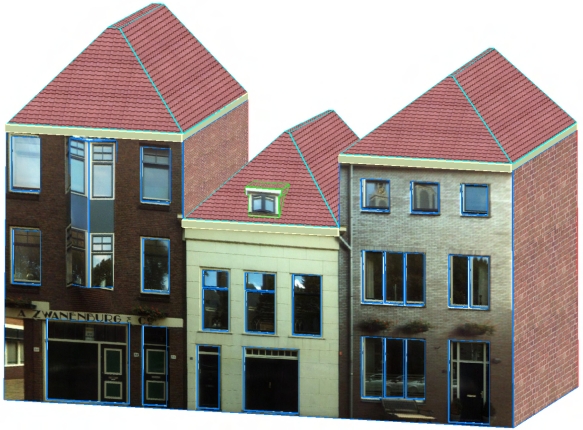
Reconstructed facade model of three joined houses.

Figure 12 shows the reconstructed facade model of the three joined houses in Figure 2. The blue lines shows the geometries of the model, which are modeled initially from laser points and then refined by strong image lines. There is a dormer on top of the left roof which is not modeled, because the density of laser points in that part is too low to recognize any features. Although this roof is visible from images (see Figure 6), the roof is still missing in the final model because images are only used to refine existing geometries. The texturing of the two-face protrusion is not accurate either. This is because the three houses' facade is considered to be coplanar when modeling the laser points, but in fact the wall of the left house is slightly curved for approximately 3 degrees. During segmentation of the laser points, this level of curvature is below the threshold for separating a new segment. However, a few pixels' offset is generated in the texturing stage when treating the curved plane as a flat plane. The texturing of the most left two windows are also not very accurate because of the curved wall plane.

### 6.2. The House with a Balcony

**Figure 13.**
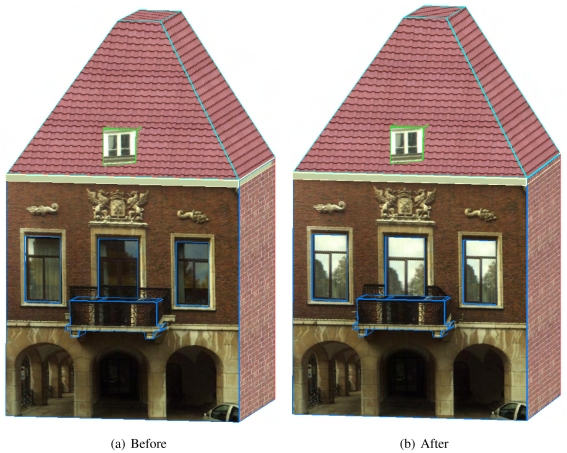
A reconstructed facade model before and after removing occluded texturing.

Figure 13 shows the reconstructed facade model of a house with a balcony on its facade. Note that the projection of the balcony remains on the wall's texture and the middle window's texture in Figure 13(a). After the wrong textured region is located, the images captured from the most left and most right locations are used for texturing alternatively (see Figure 13(b)). Unfortunately, all the image acquisition locations are below the balcony, so there is no image reaching the region which is behind and above the balcony. A part of the middle window is also inside this region. Occluded texturing of this window is avoided by replacing the actual texture with a pre-defined window image, as shown in Figure 13(b).

### 6.3. Discussion

It is realized from the test cases that accuracy of geometric modeling and image orientations are both vital to reconstructing photorealistic models. The wireframe models from only laser points are sufficient for certain purposes (cadastral management and 3D navigation for example). Decimeters even meters' inaccuracy of such building models can be tolerated, as long as the footprints coincide with 2D maps, or the general structures are recognizable together with other building models. But the accuracy requirement is much stricter when texturing is concerned, because even a few centimeter's modeling error may lead to serious color contrast near the edges. The two tested cases are both small sized buildings, and only one group of manual selected tie points are used for spatial resections. It is anticipated that covering of a large building requires more images. The accumulated error in homography estimation might be too large for the images which are faraway, and in turn leads to errors in their spatial resection. Additional manual assistance should be made to make sure that exterior orientations of all images are accurately computed. However, the required minimum distance between two manually processed images still need to be examined.

## 7. Conclusions

A promising building facade reconstruction approach by collaborating laser altimetry and optical imagery is demonstrated in this paper. Detailed building facade models with accurate geometries and realistic textures are obtained through a high automated process. It is proven that automated scene interpretation from laser data is easier than from images. As the laser points have 3D coordinates, it is straightforward to extract geometric properties like areas and surface normals. It is more difficult to extract this information from multiple (2D) images. On the other hand, feature boundaries are better extracted from imagery because of the typically higher resolution as well as the presence of mixed pixels. Besides, a lot of advanced image processing algorithms from computer vision field can be directly applied to extract building edges from images. When multiple data sources are available for a complete scene, each sub region should be processed according to its optimal reference data instead of a global reference. Laser points or images, which are acquired from a closer position and with a frontal visibility, should always be preferred.
